# Supplementary material for: Mechanical glass transition revealed by the fracture toughness of metallic glasses
Source: Nat Commun. 2018 Aug 16;9:3271. doi: 10.1038/s41467-018-05682-8 (PMC6095891; doi:10.1038/s41467-018-05682-8)
Supplement: Supplementary file 1 — Supplementary Information [file 41467_2018_5682_MOESM1_ESM.pdf]

Supplementary Information

Supplementary Figures

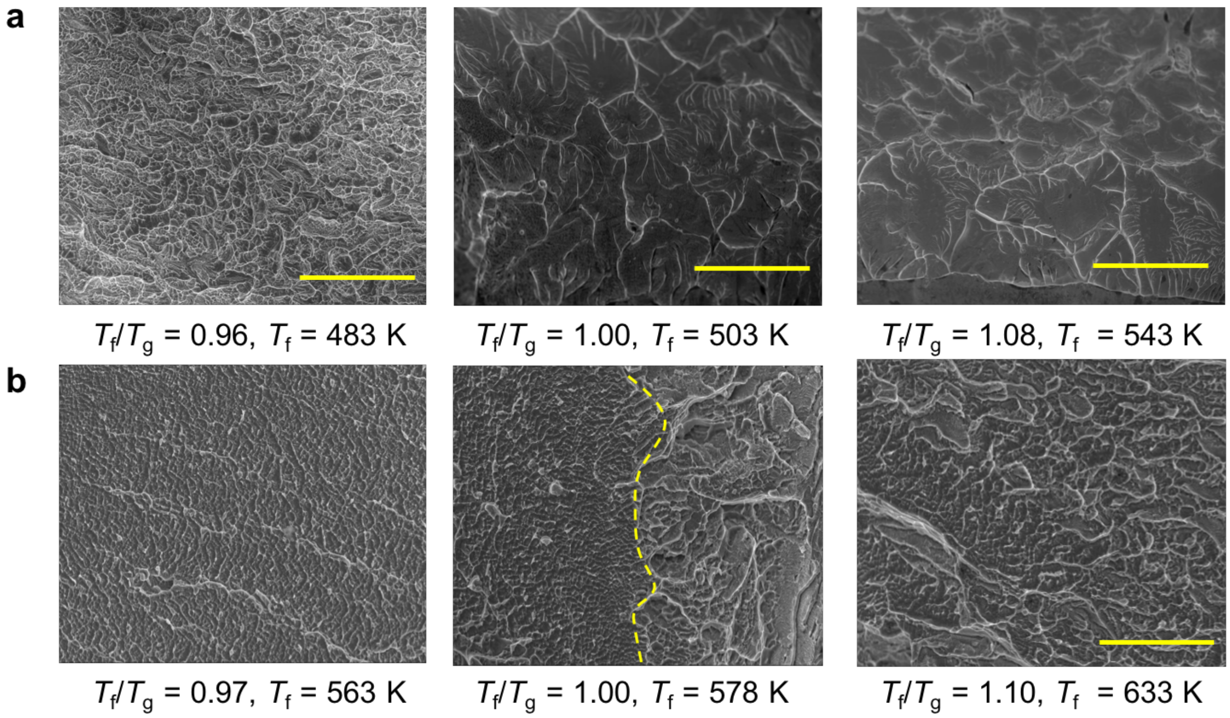

Supplementary Figure 1. Fractography of $Pd_{43}Cu_{27}Ni_{10}P_{25}$ and $Pt_{57.5}Cu_{14.7}Ni_{5.3}P_{22.5}$ for different fictive temperatures. SEM images of the fractured surface of **(a)** $Pt_{57.5}Cu_{14.7}Ni_{5.3}P_{22.5}$ and **(b)** $Pd_{43}Cu_{27}Ni_{10}P_{25}$ metallic glasses with various fictive temperatures. The scale bar for **(a)** is 50 μm and **(b)** is 20 μm. The fractography behaves similarly to that of $Zr_{44}Ti_{11}Ni_{10}Cu_{10}Be_{25}$, **(a)** $Pt_{57.5}Cu_{14.7}Ni_{5.3}P_{22.5}$ with $T_f < T_f^{DB}$ shows dimple pattern glasses above T_f^{DB} shows a ductile river pattern. **(b)** Generally, $Pd_{43}Cu_{27}Ni_{10}P_{25}$ exhibit dimple pattern as the overall K_Q (across all fictive temperatures studied here) exhibit a lower K_Q compared to the other two alloys. However, it was interesting to see that $Pd_{43}Cu_{27}Ni_{10}P_{25}$ with $T_f < T_f^{DB}$ shows significantly smaller dimple features compared to glasses with $T_f > T_f^{DB}$, while $T_f = T_f^{DB}$ shows co-existing of dimple pattern with two length scales.

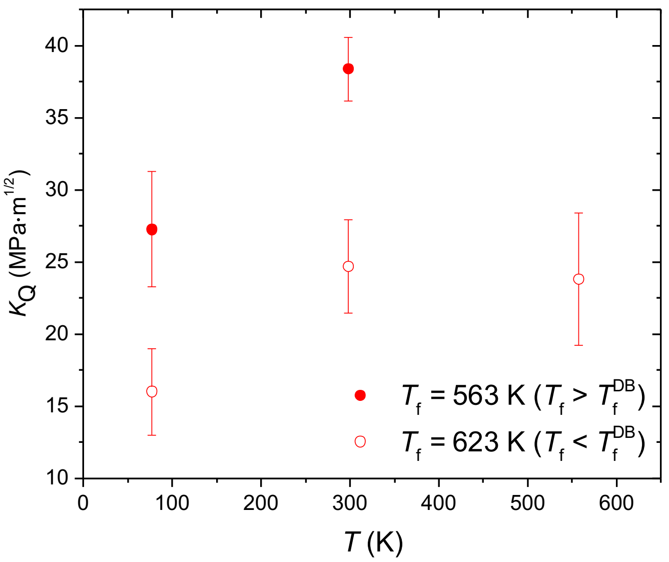

Supplementary Figure 2. K_Q of $Pd_{43}Cu_{27}Ni_{10}P_{25}$ metallic glass as a function of T. The glass exhibit gradual changes as a function of T with $T_f = 563$ ($T_f > T_f^{DB}$ - ●) and $T_f = 623$ ($T_f < T_f^{DB}$ - ○). Samples were characterized with strain rate of 10^{-4}/s. The error bars represent one standard deviation over three samples per data point.

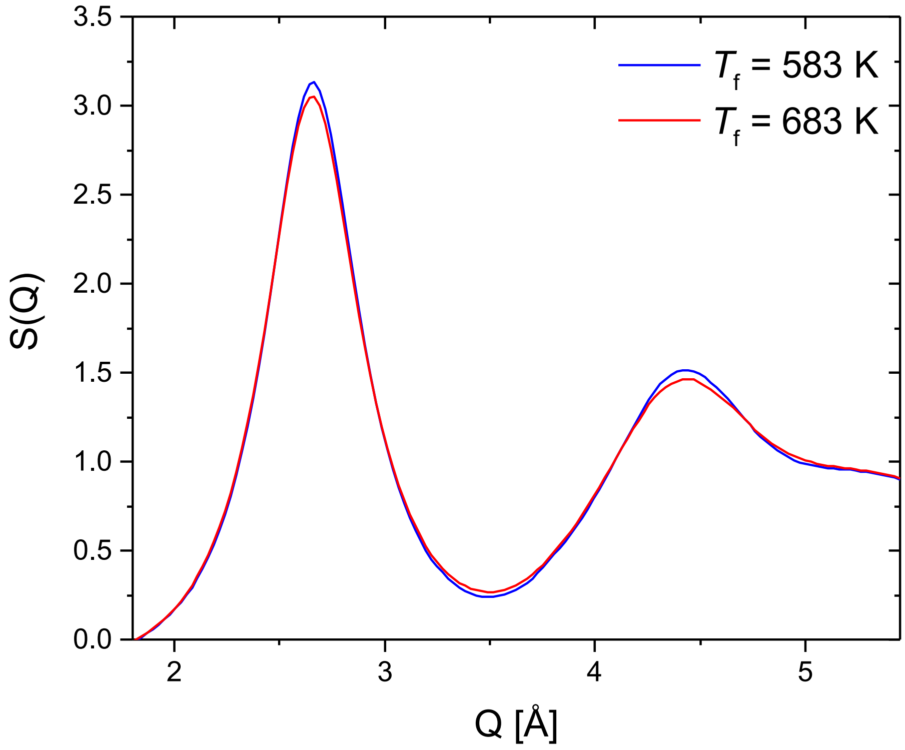

Supplementary Figure 3. The structure function, S(*Q*), for $Zr_{44}Ti_{11}Cu_{10}Ni_{10}Be_{25}$ with two extreme fictive temperatures (T_f = 583 K and 693 K). The trend indicates increase in fictive temperature results in broader S(*Q*) function.

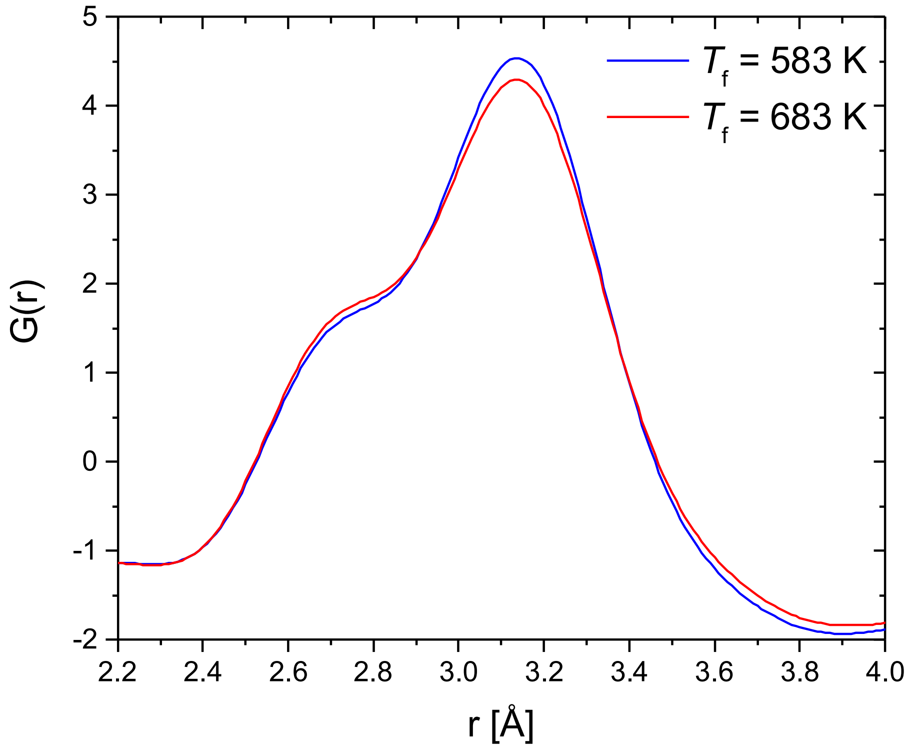

Supplementary Figure 4. The pair distribution function, *G*(*r*), for $Zr_{44}Ti_{11}Cu_{10}Ni_{10}Be_{25}$ with two extreme fictive temperatures (T_f = 583 K and 693 K). High fictive temperature results in lower amplitude and broader peak indicating more disordering as fictive temperature increases.

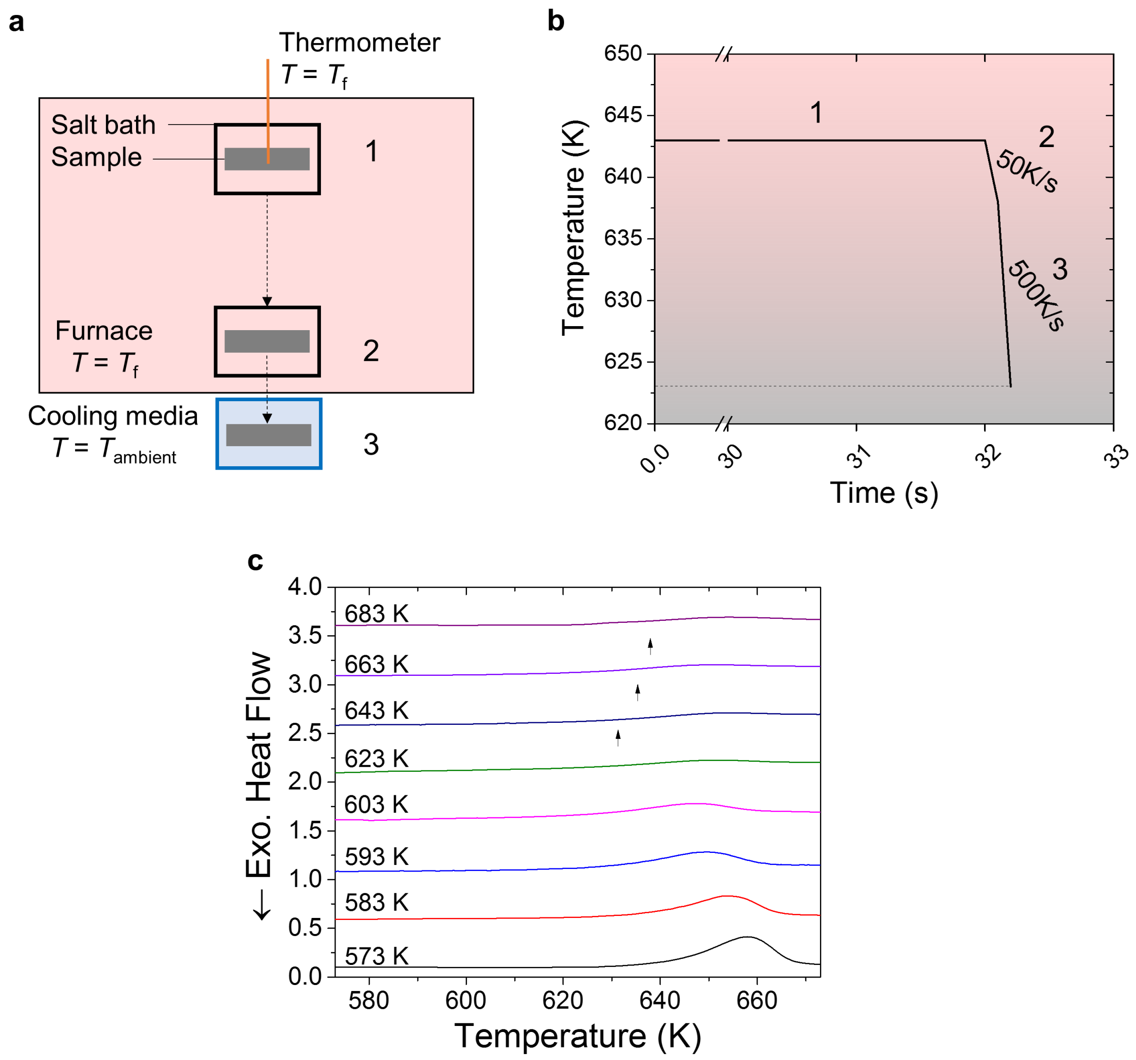

Supplementary Figure 5. Thermal history of samples and fictive temperature states. Critical for this study is that states with well-defined fictive temperatures are achieved, through full relaxation annealing at a target temperature and that the fictive temperature is conserved by fast cooling to room temperature. **(a)** The thermal history of the samples. Single-edge notched tension (SENT) specimen were fabricated by the thermoplastic forming process as described in main text. Samples were then given a specified T_f by annealing in a furnace with salt bath container where the temperature is probed by thermometer at $T = T_f$. After annealing surpasses the relaxation time required, salt bath container was moved to edge of the furnace before rapid quenching into water container of $T_{ambient}$. **(b)** shows temperature profile at point 1,2, and 3, corresponding to those labeled in (a). The temperature and time relationship is shown here is an example of $T_f = 643$ K for $Zr_{44}Ti_{11}Cu_{10}Ni_{10}Be_{25}$ (c) Differential Scanning Calorimetry thermogram showing shifting calorimetry glass transition, T_g after annealing. This verifies that glasses are relaxed into different T_f states after annealing.

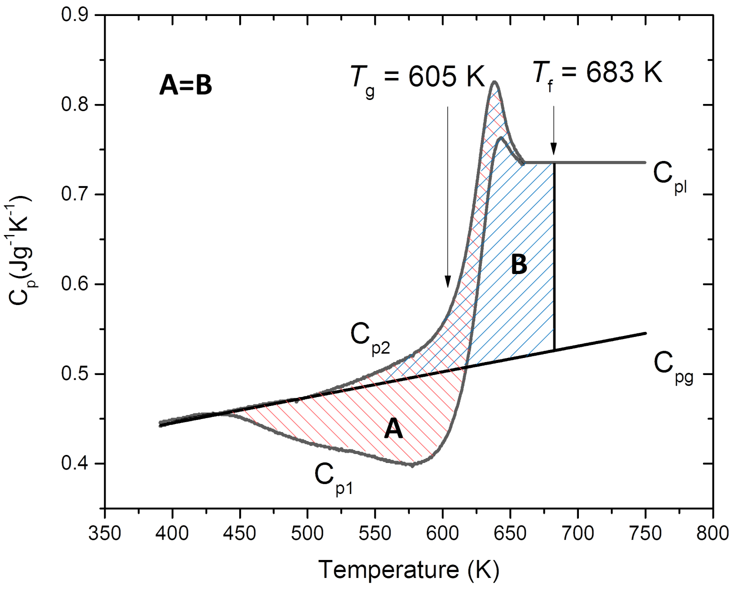

Supplementary Figure 6. Determination of fictive temperature through the energy-matching method from heat capacity measurement. $Zr_{44}Ti_{11}Cu_{10}Ni_{10}Be_{25}$ prepared through equilibrating (annealing) at 683K followed by rapid quenching to obtain a fictive temperature of 683K as described in the method section. The measurement indicate that the fictive temperature as calculated from DSC measurement indeed matches the annealing temperature (see Supplementary Method for DSC procedure).

Supplementary Method

Determination of Fictive Temperature by the Energy Matching Method

The fictive temperature can be estimated through heat capacity (C_p) measurements by the differential scanning calorimetry (DSC) (Perkin Elmer Diamond DSC). The sample was heated equilibrated at 313 K and held for 300 s, then heated to 663 K at the rate of 10 K/min and let equilibrate for 60 s, then cooled down to 573 K at 10 K/min and cooled to 313 K at 20 K/min. The procedure is repeated once. Prior to the sample measurements, the baseline was measured with an empty aluminum crucible, followed by the sapphire sample as reference. The fictive temperature was obtained following the procedure described in[1-3].

In situ structural study under uniaxial compression

MG structure under axial mechanical deformation was not isotropic, and a spherical harmonic expansion was performed to separate the anisotropic part (*l*=2, *m*=0; other high order terms are negligible) and isotropic part (*l*=0, *m*=0) from the pair-density function and structure function [4]:

$$g(r) = \sum_{l,m} g_l^m(r) Y_l^m\left(\frac{r}{r}\right), \quad S(Q) = \sum_{l,m} S_l^m(Q) Y_l^m\left(\frac{Q}{Q}\right)$$

$$g_l^m(r) = \frac{(i)^l}{2\pi^2\rho_0}\int S_l^m(Q) J_l(Qr) Q^2 dQ,$$

The number fraction of atoms participating in topological rearrangements can be calculated from the anisotropic PDF when the uniaxial stress is applied along the z-axis, $g_2^0(r)$, and is given as $|Y|$,

$$|Y| = \frac{|\Delta N_2^0|}{|N_{2,\mathrm{aff}}^0|},$$

where ΔN_2^0 is the change in the anisotropic neighbor density due to non-affine

$$\Delta N_2^0 = \pm\int_{r_0}^{r_{af}} 4\pi r^2 \rho_0 \left[g_{2,\mathrm{aff}}^0(r) - g_2^0(r)\right] dr$$

here "+" is for uniaxial tension case and "–" is for compression, r_{aff} denotes the cut-off beyond which experimental $g_2^0(r)$ agrees with the affine $g_{2,\mathrm{aff}}^0(r)$, where $g_{2,\mathrm{aff}}^0(r)$ is the anisotropic PDF expected for uniaxial affine deformation,

$$g_{2,\mathrm{aff}}^0(r) = \pm\,\varepsilon\left(\frac{1}{5}\right)^{1/2}\frac{2(1+\nu)}{3}\frac{dg_0^0(r)}{dr}$$

where ν is the Poisson's ratio, ρ_0 is the atomic number density, and ε is the elastic strain. $N_{2,\mathrm{aff}}^0$ is the anisotropic neighbor density of the affine-deformed glass obtained by integrating $g_{2,\mathrm{aff}}^0(r)$ from r_0 to r_{aff},

$$N^0_{2,\mathrm{aff}} = \int_{r_0}^{r_{af}} 4\pi r^2 \rho_0 g^0_{2,\mathrm{aff}}(r)dr,$$

Therefore, the parameter $|Y|$ reflects the heterogeneity of the elastic deformation and probes the density of the local relaxation sites in the glass created during mechanical loading.

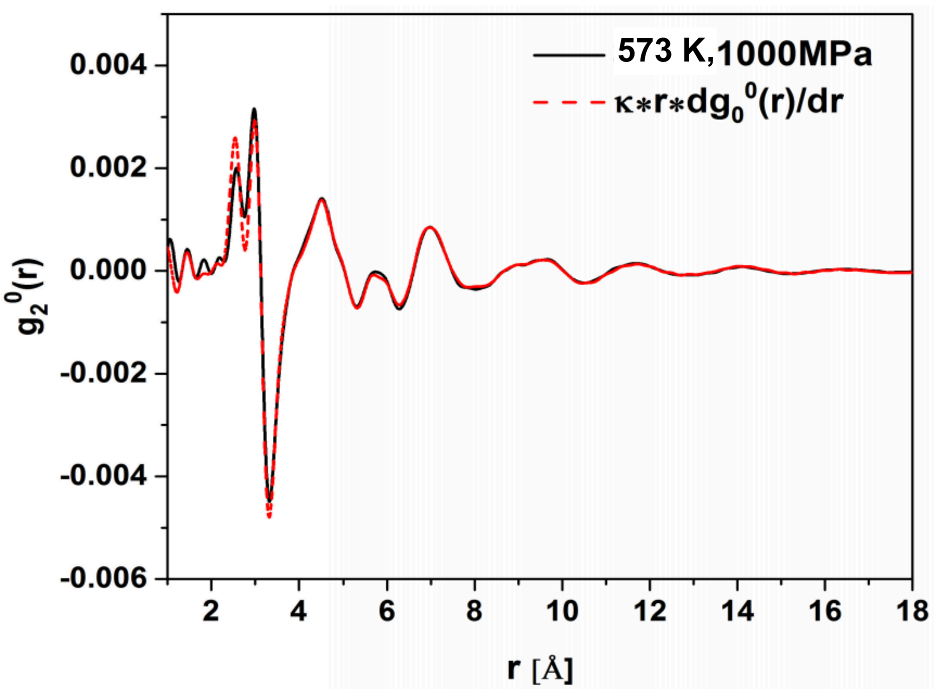

Supplementary Figure 7. show pair distribution function measured by x-ray diffraction experiment of in-situ deformation. $g^0_{2,\mathrm{affine}}(r)$ and corresponding anisotropic pair distribution function is compared in order to determine the density of the local topological rearrangements.

Supplementary References

1. Yue Y-Z. Characteristic temperatures of enthalpy relaxation in glass. Journal of Non-Crystalline Solids 354, 1112-1118 (2008).

2. Yue Y, Ohe Rvd, Jensen SL. Fictive temperature, cooling rate, and viscosity of glasses. The Journal of Chemical Physics 120, 8053-8059 (2004).

3. Yue YZ, Christiansen Jd, Jensen SL. Determination of the fictive temperature for a hyperquenched glass. Chemical Physics Letters 357, 20-24 (2002).

4. Dmowski W, Iwashita T, Chuang CP, Almer J, Egami T. Elastic heterogeneity in metallic glasses. Phys Rev Lett 105, 205502 (2010).
